# OGG1-initiated base excision repair exacerbates oxidative stress-induced parthanatos

**DOI:** 10.1038/s41419-018-0680-0

**Published:** 2018-05-24

**Authors:** Ruoxi Wang, Chunshuang Li, Ping Qiao, Yaoyao Xue, Xu Zheng, Hongyu Chen, Xianlu Zeng, Wenguang Liu, Istvan Boldogh, Xueqing Ba

**Affiliations:** 1 The Key Laboratory of Molecular Epigenetics of Ministry of Education, Northeast Normal University, Changchun, Jilin 130024 China; 2 School of Life Science, Northeast Normal University, Changchun, Jilin 130024 China; 3 Department of Cell Biology, College of Basic Medical Sciences, Jilin University, Changchun, 130021 China; 4 Department of Microbiology and Immunology, University of Texas Medical Branch at Galveston, Galveston, TX 77555 USA; 5 Sealy Center for Molecular Medicine, University of Texas Medical Branch at Galveston, Galveston, TX 77555 USA

## Abstract

Oxidative stress-induced DNA damage has been well acknowledged as a major cause leading to cell death, which is etiologically linked to ischemic injury and degenerative alterations. The most common oxidation product of DNA is base lesion 8-oxo-7,8-dihydroguanine (8-oxoG), which is repaired by 8-oxoG glycosylase1 (OGG1)-initiated baseexcision repair (BER) pathway (OGG1-BER); however, the role of OGG1-BER in oxidative stress-induced cell death is poorly investigated. DNA strand breaks and apurinic/apyrimidinic (AP) sites are effective substrates to activate DNA damage sensor poly(ADP-ribose) polymerase 1 (PARP1). Overactivation of PARP1 is associated with apoptosis-inducing factor (AIF)-mediated and caspase-independent cell death (parthanatos). We hypothesized that after an excessive oxidative insult, OGG1-BER-generated strand breaks result in hyperactivation of PARP1 and consequently cell death. To test, wild type, knockout, siRNA-depleted MEFs and neuroblastoma cells, or those expressing repair-deficient OGG1 mutants were oxidatively stressed and the role of OGG1 was examined. Results showed that OGG1-BER further increases the levels of ROS-induced DNA damage by generating repair intermediates, leading to PARP1 overactivation and cell death. Cells lacking or expressing repair-deficient OGG1 showed lower levels of DNA strand lesions, PARP1 activation, and nuclear translocation of apoptosis-inducing factor, resulting in the increased resistance to ROS-induced parthanatos. These results suggested that OGG1 guards genome integrity through either lesion repair or elimination of cells with malignant potential, to maintain the homeostasis of the host, which might depend on the magnitude of guanine oxidation.

## Introduction

Oxidative stress is referred to elevated intracellular level of reactive oxygen species (ROS) that inevitably derive from various endogenous physiological processes, which can be exacerbated by environmental exposures^1^. ROS cause oxidative damage of lipids, proteins, and DNA, and to maintain genome integrity, DNA lesions ought to be repaired^1,2^. In the genomic DNA, one of the most common oxidation products is base lesion 8-oxo-7,8-dihydroguanine (8-oxoG), which is one of the best biomarker of oxidative stress^3,4^. 8-OxoG is mutagenic, and the cognate enzyme that specifically recognizes and repairs 8-oxoG and its open-ring product 2,6-diamino-4-hydroxy-5-formamidopyrimidine (FapyG) is 8-oxoguanine DNA glycosylase 1 (OGG1), a functional homolog of *Escherichia coli* protein MutM^5,6^. Base excision repair (BER) is a multistep process, which is described as a “hand-off” model, including lesion recognition, base excision, and strand cleavage, followed by recruitment of apurinic/apyrimidinic (AP) endonuclease 1 (APE1), DNA pol β, and DNA ligase III to the presumptive scaffold protein X-ray repair cross-complementing 1 (XRCC1) to complete the repair process^7–9^.

Oxidative stress-induced DNA damage has been well acknowledged as a major cause leading to cell death, which is etiologically linked to ischemic injury and degenerative alterations^10,11^. However, the role of OGG1-BER in oxidative stress-induced cell death is poorly investigated. Dr. Dawson’s group documented a distinct mode of cell death, namely, parthanatos, which is refered to as PARP1-dependent, apoptosis-inducing factor (AIF)-mediated and caspase-independent cell death^12,13^. Unlike typical apoptosis, parthanatos does not cause apoptotic body formation or small-scale DNA fragmentation. When apoptosis causes phosphatidylserine flipping onto the outer plasma membrane and manifests propidium iodide (PI) exclusion, parthanatos exhibits both annexin-V labeling and PI staining as positive^14^. As a DNA damage sensor, PARP1 can be activated via binding to both DNA breaks and AP sites^15,16^, and upon activation, PARP1 catalyzes the formation of polymerized ADP-ribose (PAR) from nicotinamide adenine dinucleotide (NAD^+^), and subsequently the covalent attachment of PAR polymers to target proteins. In turn, PAR polymer is removed from the target proteins by successively activated PAR glycohydrolase (PARG), forming the free PAR^17^. Acting as death messenger, free PAR is mainly produced in the nucleus and then redistributed to the cytoplasm and mitochondria, where it is critical for the release of apoptosis-inducing factor (AIF) from mitochondria and then its translocation into the nucleus^18^. AIF induces chromatin condensation and large-scale DNA fragmentation (~50 kb) leading to cell death^12,13,18^.

Parthanatos is linked to diseases including stroke, Parkinson’s disease, heart attack, diabetes, and ischemia reperfusion injury^19,20^, where the intracellular context is well-acknowledged to be highly perturbed by ROS, and guanines are supposed to be excessively oxidized^4,21^. Studies have documented that excitotoxic activation of *N*-methyl-d-aspartate (NMDA) receptor results in formation of ROS, which causes DNA damage, thereby the excessive PARP1 activation and AIF-mediated cell death (parthanatos)^14,22^. It has been proposed that processing of oxidatively generated clustered DNA lesions may be prolonged, which leads to the increase in the persistent strand breaks^23^. Given that AP sites and strand breaks are the intermediates of OGG1-BER, we questioned whether OGG1-initiated base-excision repair plays a role in PARP1-driven parthanatos. Our study showed that OGG1 expression increased, while genetic or siRNA-mediated ablation of *Ogg1*, as well as expression of enzymatically deficient OGG1 decreased accumulation of DNA strand breaks, PARP1 overactivation, and AIF nuclear translocation, and consequently, cell death. We also documented OGG1’s role in NMDA-induced parthanatos in SH-SY5Y neuroblastoma cells. The present study indicated that excessive OGG1-BER could be lethal for cells subjected to a high level of oxidative insult.

## Results

### OGG1 exacerbates H_2_O_2_-induced PARP1-dependent AIF-induced cell death

**Fig. 1:**
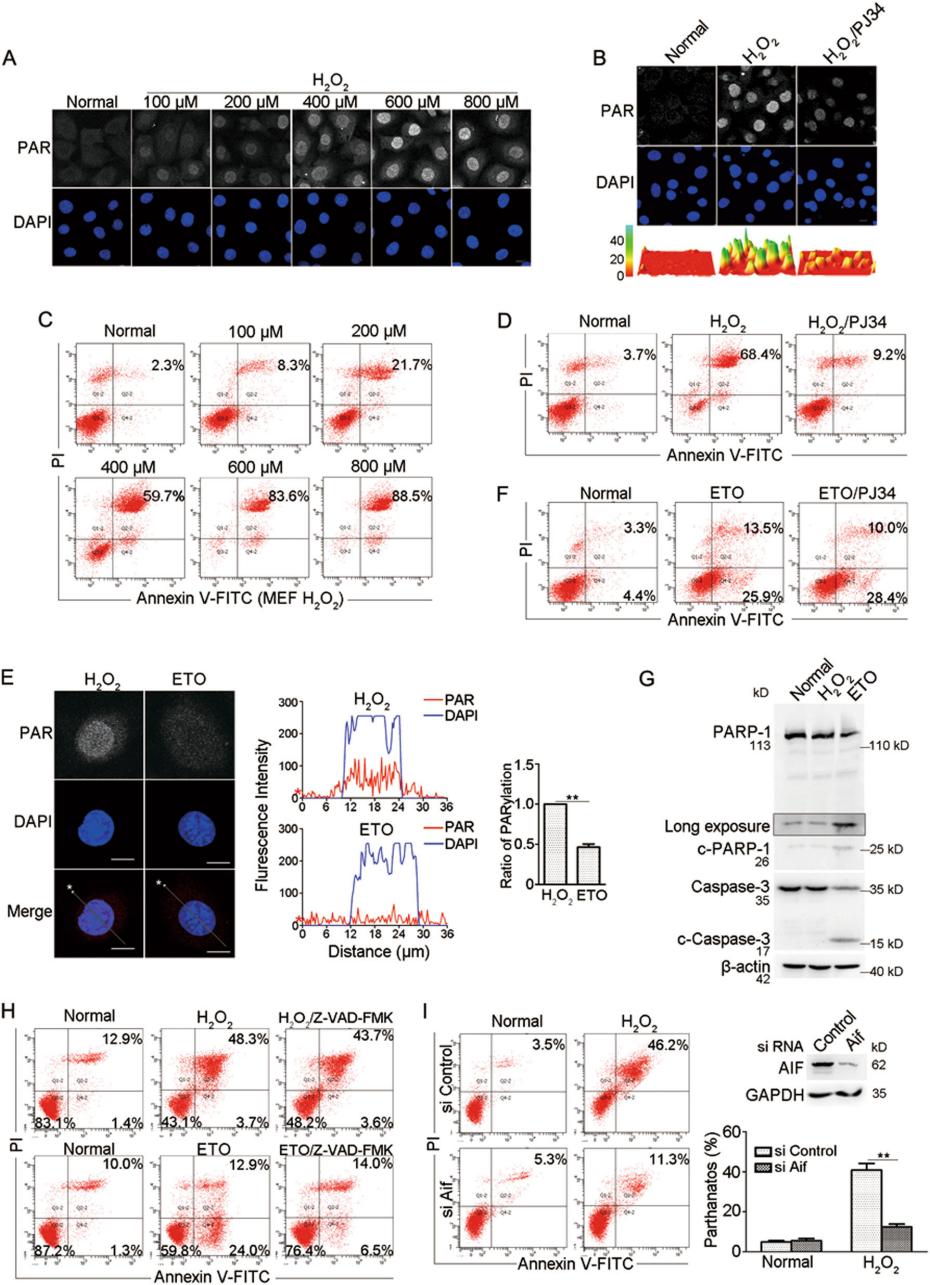
Oxidative stress induces cells undergoing typical parthanatos. **a** Microscopic assessment of protein PARylation in cells exposed to H_2_O_2_. MEFs were incubated with increasing concentrations of H_2_O_2_ for 30 min. Immunofluorescence microscopy was performed to visualize PAR signals. Nuclei were counterstained with DAPI. Scale bar: 10 μm. **b** PJ34 inhibits H_2_O_2_-induced PARP1 activation. MEFs were incubated with 400 μM H_2_O_2_ in the presence of 2.5 μM PJ34 or not. Immunofluorescence microscopy was conducted to analyze PARP1 activation. The lower row shows the three-dimensional plot of the intensity of PARylation shown in the upper panels, as determined by using Image J software. Scale bar: 10 μm. **c** H_2_O_2_ exposure induces cell death in a dose-dependent manner. MEFs were incubated with increasing concentrations of H_2_O_2_ for 24 h. Flow cytometry analysis of annexin V-FITC/PI staining was conducted to examine cell death. **d** PARP1 activation is involved in cell death induced by H_2_O_2_. MEFs were incubated with 400 μM H_2_O_2_ for 24 h in the presence of 2.5 μM PJ34 or not. Flow cytometry analysis of annexin V-FITC/PI staining was conducted to examine cell death. **e**, **f** ETO fails to elicit typical PARP1 activation. **e** MEFs were incubated with 400 μM H_2_O_2_ or 300 μM ETO for 30 min. Immunofluorescence microscopy was conducted to visualize PAR signals. Nuclei were counterstained with DAPI. The middle panel shows the fluorescence intensity of PARylation from line scans in the merged images, analyzed by Image Pro Plus software (red line: PARlytion; blue line: DAPI). The right panel shows the relative fluorescence intensity of Parylation with an average of 100 cells counted and analyzed by Image J software. Scale bar: 10 μm. **p < 0.01. And **(f)** MEFs were incubated with 300 μM ETO for 24 h in the presence of 2.5 μM PJ34 or not. Flow cytometry analysis of annexin V-FITC/PI staining was conducted to examine cell death. **g** Treatment of ETO but not H_2_O_2_ induces the cleavage of PARP1 and caspase-3. MEFs were incubated with 400 μM H_2_O_2_ or 300 μM ETO for 24 h. Whole-cell lysates were prepared, and western blotting was performed to determine the cleavage of PARP1 and caspase-3. **h** H_2_O_2_-induced cell death is caspase-independent. MEFs were incubated with 400 μM H_2_O_2_ or 300 μM ETO for 24 h in the presence of pan-caspase inhibitor (Z-VAD.FMK; 100 μM) or not. Flow cytometry analysis of annexin V-FITC/PI staining was conducted to examine cell death. **i** Knockdown of AIF decelerates cell death induced by H_2_O_2_ exposure. MEFs were transfected with siRNA targeting *Aif* or control for 36 h, and then incubated with H_2_O_2_ (400 μM) for 24 h. Flow cytometry analysis of annexin V-FITC/PI staining was conducted to examine cell death (left). The right panel shows the efficiency of AIF interference (upper) and the quantifications of annexin V and PI double-positive cells (lower). **p < 0.01, *n* ≥ 3

Although it is not a free radical, hydrogen peroxide (H_2_O_2_) has a relative long lifespan, may travel long distances within cells, and be efficiently converted into hydroxyl radical, efficacious to activate PARP1;^24,25^ thus we used H_2_O_2_ to mimic oxidative stress. Incubation of MEFs with H_2_O_2_ for 30 min induced protein PARylation in a dose-dependent manner. An overt increase in the level of PARylated proteins was observed upon 400 μM H_2_O_2_ exposure (Fig. 1a; Fig. S1A), which was diminished by PARP1 inhibitor PJ34 (Fig. 1b; Fig. S1B). Flow cytometry analysis showed that H_2_O_2_ exposure also induced cell death in a dose-dependent manner (Fig. 1c). Incubation with 400 μM H_2_O_2_ resulted in ~60% annexin V and PI dual-positive cells, which was markedly suppressed by PJ34 to <10% (Fig. 1d). As control, etoposide (ETO) was utilized. ETO is widely used for chemotherapy that introduces DNA double-strand breaks (DSBs) by inhibition of top II, leading to cells undergoing typical apoptosis^26^. Flow cytometry analysis showed that ETO exposure triggered cell death in a dose-dependent manner (Fig. S2A). However, incubation of MEFs with ETO for 30 min did not induce a typical PARP1 activation regarding the magnitude and the location of the PARylation signals (Fig 1e; Fig. S2B). While H_2_O_2_-induced PARylation majorly emerged from the nucleus, forming the signal foci, ETO-induced PARylation was weak and evenly distributed both in the cytoplasm and nucleus (Fig. 1e). Nevertheless, PJ34 could not suppress ETO-induced cell death, which primarily exhibited as annexin V positive and PI negative (Fig. 1f). In parallel, cleavage of caspase-3 and PARP1, the typical marks of apoptosis were induced by ETO but not H_2_O_2_ exposure (Fig. 1g), and caspase-3 activity was hardly detectable during the timescale of H_2_O_2_ exposure (Fig. S3). The broad-spectrum caspase inhibitor (caspase-inhibitor VI; Z-VAD-FMK) failed to block H_2_O_2_-induced increase in annexin V and PI dual-positive cells, but effectively inhibited ETO-induced increase in annexin-V-positive and PI-negative cells (Fig. 1h). To further prove that the H_2_O_2_-induced cell death is AIF-mediated, MEFs were transfected with siRNA against *Aif* or control, and then incubated with H_2_O_2_. Flow cytometry indicated that the percentage of annexin V and PI dual-positive cells was significantly decreased by siAIF (Fig. 1i). Data suggested that oxidative stress-induced cell death is parthanatos one.

**Fig. 2:**
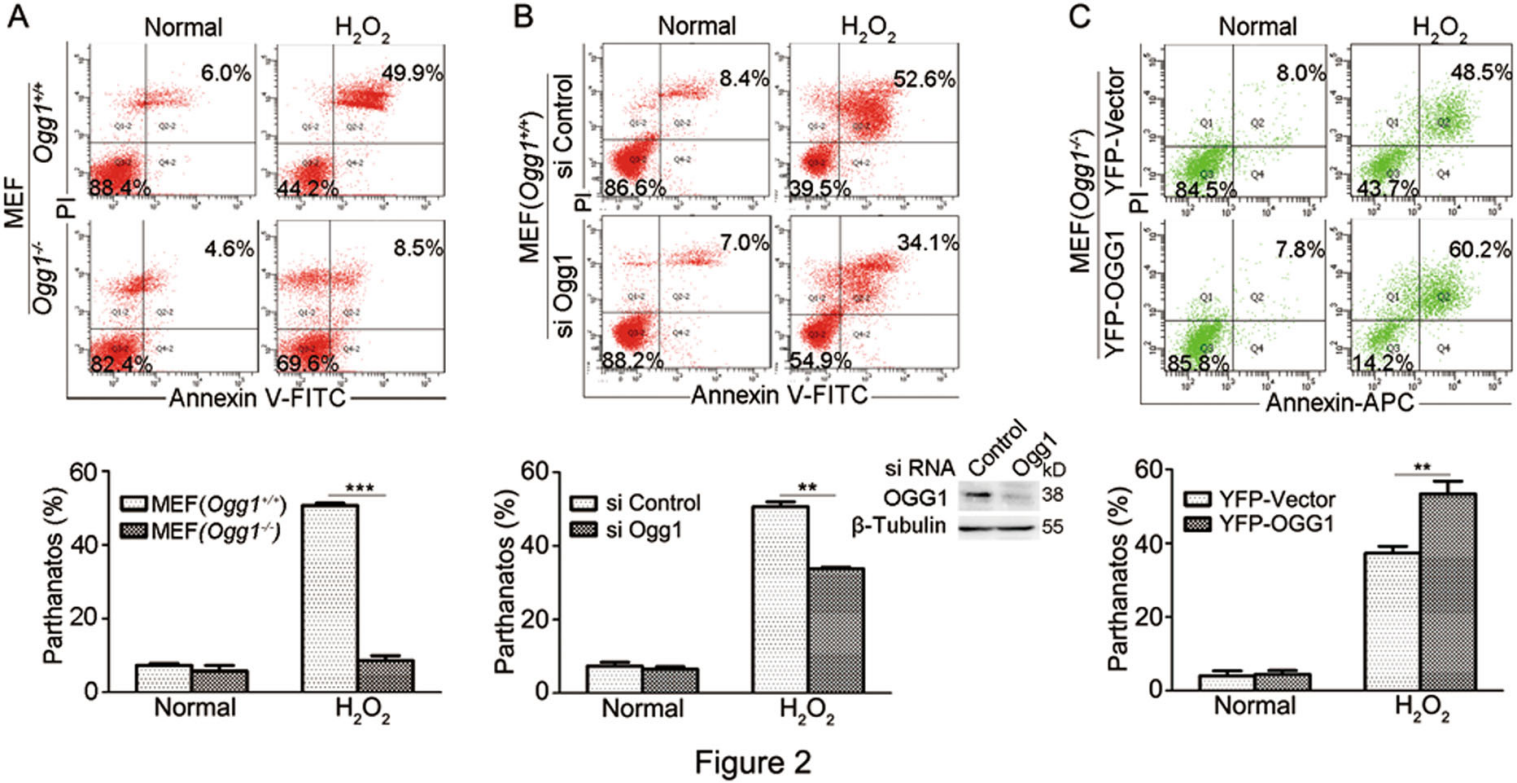
OGG1 exacerbates H_2_O_2_-induced cell parthanatos. **a** *Ogg1*^*+/+*^ MEFs are more vulnerable to undergo parthanatos than *Ogg1*^−/−^ MEFs upon H_2_O_2_ exposure. *Ogg1*^*+/+*^ and *Ogg1*^*−/−*^ MEFs were incubated with 400 μM H_2_O_2_ for 24 h. Flow cytometry analysis of annexin V-FITC/PI staining was conducted to examine cell death (upper). **b** Knockdown of Ogg1 ameliorates cell death induced by H_2_O_2_ exposure. MEFs were transfected with siRNA targeting *Ogg1* or control for 36 h, and then incubated with H_2_O_2_ (400 μM) for 24 h. Flow cytometry analysis of annexin V-FITC/PI staining was conducted to examine cell death (upper). **c** Overexpression of OGG1 enhances H_2_O_2_-induced cell death. YFP-OGG1 and control YFP-vector plasmids were transfected into *Ogg1*^*–/–*^ MEFs for 36 h, and then incubated with H_2_O_2_ (400 μM) for 24 h. The YFP-positive cells were gated out, and H_2_O_2_-induced cell death was analyzed by flow cytometry analysis of annexin V-APC/PI staining (upper). Lower panels show the quantifications of annexin V and PI double-positive cells shown in the upper panels (*n* ≥ 3) as well as the efficiency of Ogg1 interference. **p < 0.01, ***p < 0.001

Next, we tested whether OGG1 expression affects H_2_O_2_-induced cell death. Wild-type (*Ogg1*^*+/+*^) and *Ogg1* knockout (*Ogg1*^*–/*^^–^) MEFs cells were exposed to 400 μM H_2_O_2_ for 24 h. Flow cytometry showed that the percentage of annexin V and PI double-positive *Ogg1*^*+/+*^ MEFs was almost sixfold higher than that of *Ogg1*^*−/−*^ MEFs (Fig. 2a). In parallel, knockout of *endonuclease III-like protein I* (*Nth1*), the BER enzyme specially repairing thymine glycol, reduced H_2_O_2_-induced parthanatos to ~50% (Fig. S4), suggesting that OGG1 expression is more decisive in oxidative stress-induced cell death. To further affirm the effect of OGG1, MEFs were transfected with siRNA against *Ogg1* or control, and then incubated with H_2_O_2_. Flow cytometry indicated that the percentage of cell death was significantly decreased by siOgg1 (Fig. 2b). Furthermore, YFP-OGG1 and the YFP-vector plasmids were transfected into *Ogg1*^*−/−*^ MEFs. The YFP-positive cells were gated out and H_2_O_2_-induced cell death was analyzed. Results showed that *Ogg1*^*−/−*^ MEFs with YFP-OGG1 overexpression underwent more severe cell death than the cells expressing YFP alone did (Fig. 2c). The combined data demonstrated that OGG1 expression exacerbated oxidative stress-induced cell parthanatos.

### OGG1 is implicated in the activation of parthanatos signal pathway in oxidatively stressed cells

**Fig. 3:**
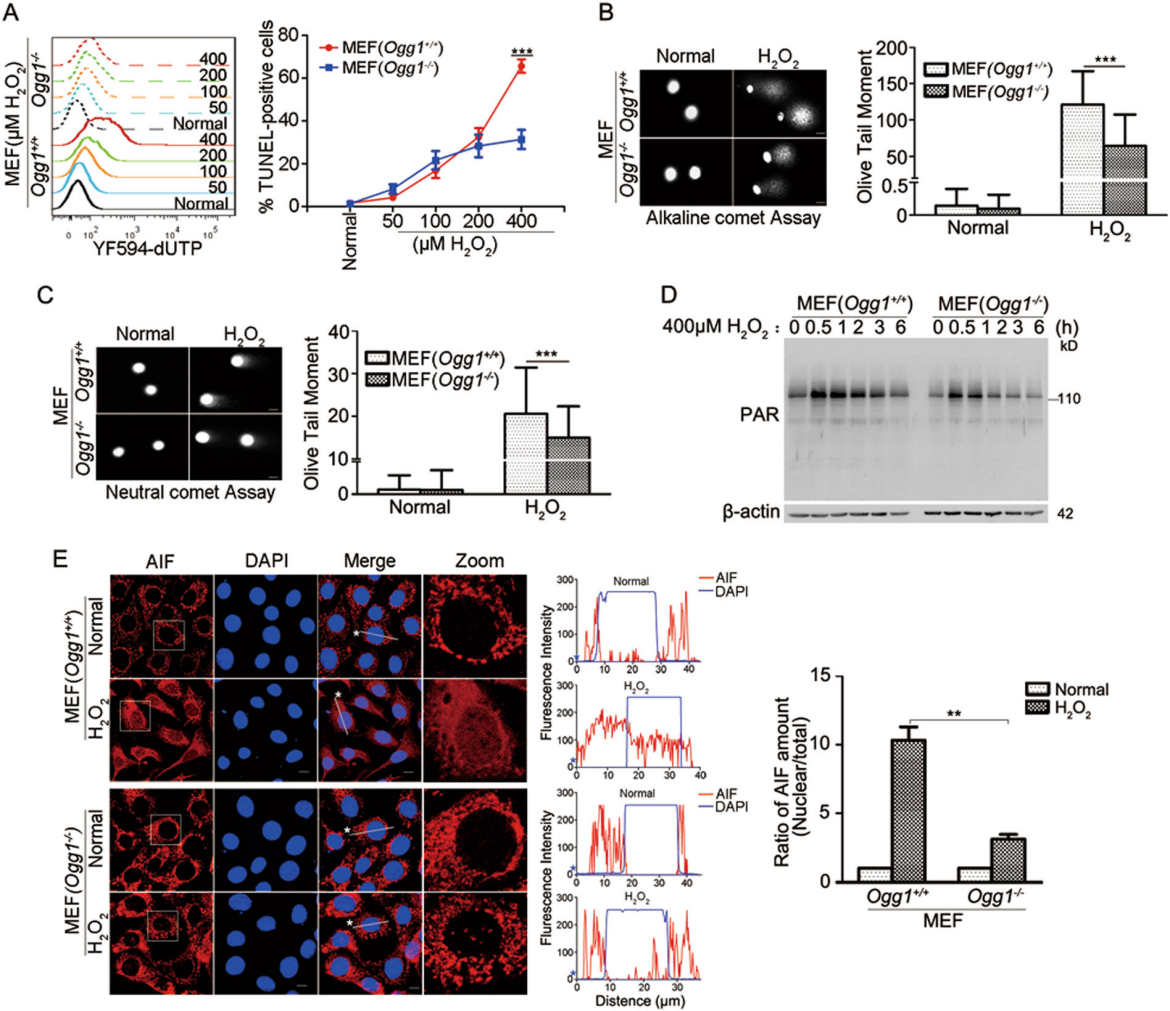
OGG1 promotes the activation of parthanatos signal pathway in cells exposed to H_2_O_2_. **a** High level of H_2_O_2_ induces more DNA strand breaks in cells expressing Ogg1 than that in *Ogg1* knockout cells. *Ogg1*^*+/+*^ and *Ogg1*^*−/−*^ MEFs were incubated with increasing concentrations of H_2_O_2_ for 3 h, and TUNEL assay was conducted. Cells were stained for detection of 3′-OH DNA termini, as described under Materials and methods and analyzed by flow cytometry (left). Percentages of TUNEL-positive cells were quantified (right). *n* ≥ 3. **b**, **c** Analyses of DNA strand breaks in cells exposed to H_2_O_2_ by comet assays. *Ogg1*^*+/+*^ and *Ogg1*^*−/−*^ MEFs were incubated with 400 μM H_2_O_2_ for 30 min. **b** Alkaline comet assay was conducted to analyze both SSBs and DSBs, and **(c)** neutral comet assay to analyze DSBs, scale bar: 10 μm. The olive tail moment was determined by using Cometscore software. One hundred individual comets were counted for each sample (left panels). **d** PARP1 activation is stronger in Ogg1-expressing cells upon exposure of H_2_O_2_. *Ogg1*^*+/+*^ and *Ogg1*^*−/−*^ MEFs were exposed to H_2_O_2_ for the increasing lengths of time as indicated. Western blotting was conducted to analyze the levels of protein PARylation. **e** OGG1 promotes translocation of AIF to the nucleus in response to H_2_O_2_ exposure. *Ogg1*^*+/+*^ and *Ogg1*^*−/−*^ MEFs were incubated with H_2_O_2_ for 12 h, and then, the cells were fixed and labeled with an antibody against AIF. The nuclei were counterstained with DAPI. The right column of the left panel shows magnified views of the boxed area in the AIF images. The profiles in the middle panel show the fluorescence intensity of AIF from line scans in the merged images, analyzed by Image Pro Plus software (red line: AIF; blue line: DAPI). Quantitative analysis of AIF nuclear transfer is shown in the right panel, with an average of 100 cells counted (for image analysis method see Materials and methods). Scale bar: 10 μm. **p < 0.01, ***p < 0.001

To determine whether OGG1 is the causative factor for the increase in DNA strand breaks that enhances the activation of PARP1, TUNEL assay was conducted, and DNA breaks induced by increasing levels of H_2_O_2_ were examined. TUNEL-positive Ogg1-expressing and Ogg1-null cells showed no significant difference along with the increase in H_2_O_2_ concentrations up to 200 µM. However, when cells were challenged with 400 µM H_2_O_2_, the number of TUNEL-positive Ogg1-expressing cells was twofold more than that of Ogg1-null cells (Fig. 3a). Data suggested that if oxidative stress is moderate, OGG1 may repair the lesion effectively and efficiently, and DNA breaks may come from the direct attack of ROS to DNA backbone; whereas, OGG1-BER may unexpectedly become a major source of DNA strand breaks in response to an aggressive ROS insult. Next, we performed comet assays under different conditions. Alkaline comet assay, which detects DNA breaks including both DSBs and SSBs, revealed that H_2_O_2_-treated *Ogg1*^*+/+*^ MEFs have more DNA strand breaks compared with *Ogg1*^*−/−*^ MEFs (Fig. 3b). Neutral comet assay, by which only DSBs are detectable, also showed that H_2_O_2_-treated *Ogg1*^*+/+*^ MEFs have more double DNA strand breaks (Fig. 3c). Phosphorylation of ser139 residue of H2AX (γH2AX) is a mark of DSBs. In support, western blotting indicated a rapid increase in γH2AX content in *Ogg1*^*+/+*^ MEFs, which retained at the high level from 2 to 6 h post H_2_O_2_ exposure; whereas, *Ogg1*^*−/−*^ MEFs showed less H2AX phosphorylation and the level of γH2AX gradually decreased after 2 h of H_2_O_2_ addition (Fig. S5A).

Accordingly, as shown by the formation of PAR polymers, PARP1 activation was elicited at 30 min, and reversed down from 2 h, and went back to the basal level at 6 h after addition of H_2_O_2_ in *Ogg1*^*+/+*^ MEFs; whereas in *Ogg1*^*−/−*^ MEFs, the level of protein PARylation was markedly lower, and normalized after 3 h of H_2_O_2_ exposure (Fig. 3d). Moreover, immunofluorescence microscopy showed that nuclear AIF signals were overt in *Ogg1*^*+/+*^ MEFs compared with that in the *Ogg1*^*−/−*^ MEFs (Fig. 3e). This result was confirmed by western blot (Fig. S5B). Taken together, these data suggested that OGG1 increases oxidative stress-induced DNA strand breaks, which led to the hyperactivation of PARP1 and the translocation of AIF to the nucleus.

### OGG1’s BER activity accounts for oxidative stress-induced parthanatos

**Fig. 4:**
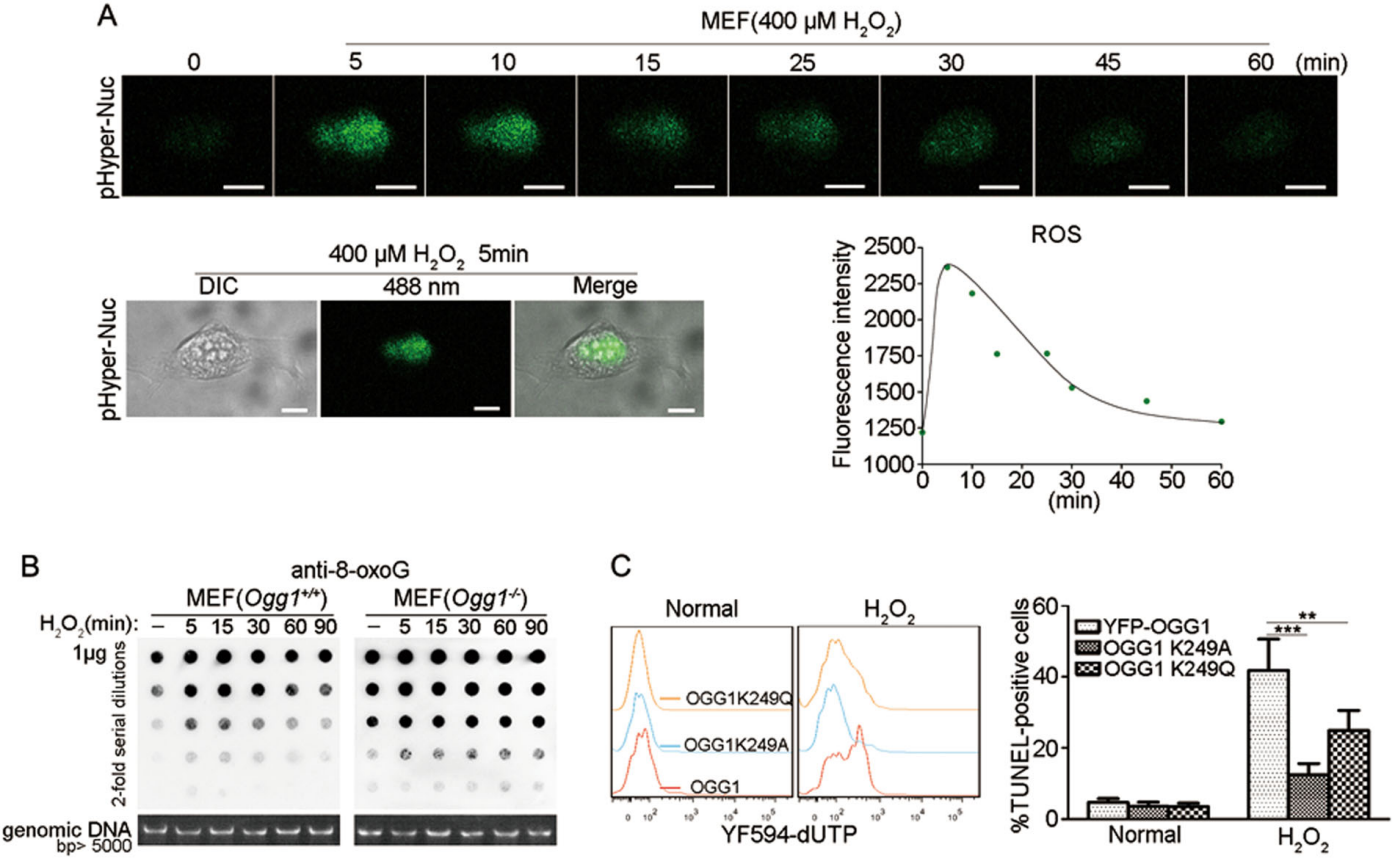
OGG1’s BER activity enhances H_2_O_2_-induced DNA strand breaks. **a** The kinetic changes in the nuclear ROS level in MEF cells after H_2_O_2_ exposure. Plasmids pHyper-Nuc were transfected into MEFs for 48 h, and then treated with H_2_O_2_ (400 μM). The sequential images were captured by spinning-disk confocal microscope. Scale bar: 10 μm. Upper: the kinetic changes in the nuclear ROS level; left of lower: the distribution of the nuclear ROS sensor pHyPer-Nuc GFP; right of lower: quantification of the kinetics of the nuclear ROS level by Volocity 5.5.1 software. **b** Impaired 8-oxoG repair in *Ogg1*-null cells upon H_2_O_2_ exposure. *Ogg1*^*+/+*^ and *Ogg1*^*−/−*^ MEFs were incubated with H_2_O_2_ for increasing lengths of time as indicated. Genomic DNA was isolated and global 8-oxoG levels were analyzed by dot-blot assay using 8-oxoG antibody as described under Materials and methods. **c** Functional defect of OGG1 ameliorates DNA strand breaks in cells upon H_2_O_2_ exposure. YFP-OGG1, YFP-OGG1K249A, and YFP-OGG1K249Q plasmids were transfected into *Ogg1*^*−/−*^ MEFs for 36 h, and then incubated with H_2_O_2_ (400 μM) for 6 h. Cells were stained for detection of 3′-OH DNA termini (TUNEL assay) and the YFP-positive cells were gated out and analyzed by flow cytometry, *n* ≥ 3; **p < 0.01, ***p < 0.001

We explored whether OGG1-BER exacerbates oxidative stress-induced DNA strand break formation and PARP1 activation. To this end, nuclear ROS level and genomic 8-oxoG content were monitored first. The nuclear ROS sensor pHyPer-Nuc GFP was overexpressed in MEFs, and after addition of H_2_O_2_ in a medium, the ROS level presented by the intensity of GFP fluorescence reached the summit at 5 min and then gradually decreased to basal level by 60 min (Fig. 4a). Likewise, the kinetics of pHyper-Nuc GFP fluorescence in *Ogg1*^*−/−*^ MEFs showed the same changes (data not shown). Accordingly, dot-blot analysis showed that H_2_O_2_ exposure led to a global increase in genomic 8-oxoG level in *Ogg1*^*+/+*^ MEF cells from 5 min onward, which peaked at 15 min, and then fell back progressively; whereas the increase in 8-oxoG level in *Ogg1*^*−/−*^ MEF cells persisted from 5 min onward to over 90 min (Fig. 4b). The result not only indicated an increase in 8-oxoG formation upon the robust elevation of nuclear ROS level, as well as a permissible repair of the guanine lesions in the OGG1-expressing cells along with the restoration of the redox balance, but also implied that the high level of 8-oxoG itself may not increase the fatality.

To verify that OGG1-BER causes PARP1 overactivation and contributes to oxidative stress-induced parthanatos, YFP-OGG1 and the inactive mutants YFP-OGG1K249A, OGG1K249Q plasmids were constructed and transfected in *Ogg1*^*−/−*^ MEFs. The residue lysine 249 in OGG1 active site is crucial for its DNA glycosylase and AP lyase activities^27^. Accordingly, TUNEL labeling showed that YFP-OGG1-expressing cells had a significantly higher level of DNA strand breaks (~40%) than YFP-OGG1K249A- (~12%) and OGG1K249Q-expressing (~24%) cells (Fig. 4c).

**Fig. 5:**
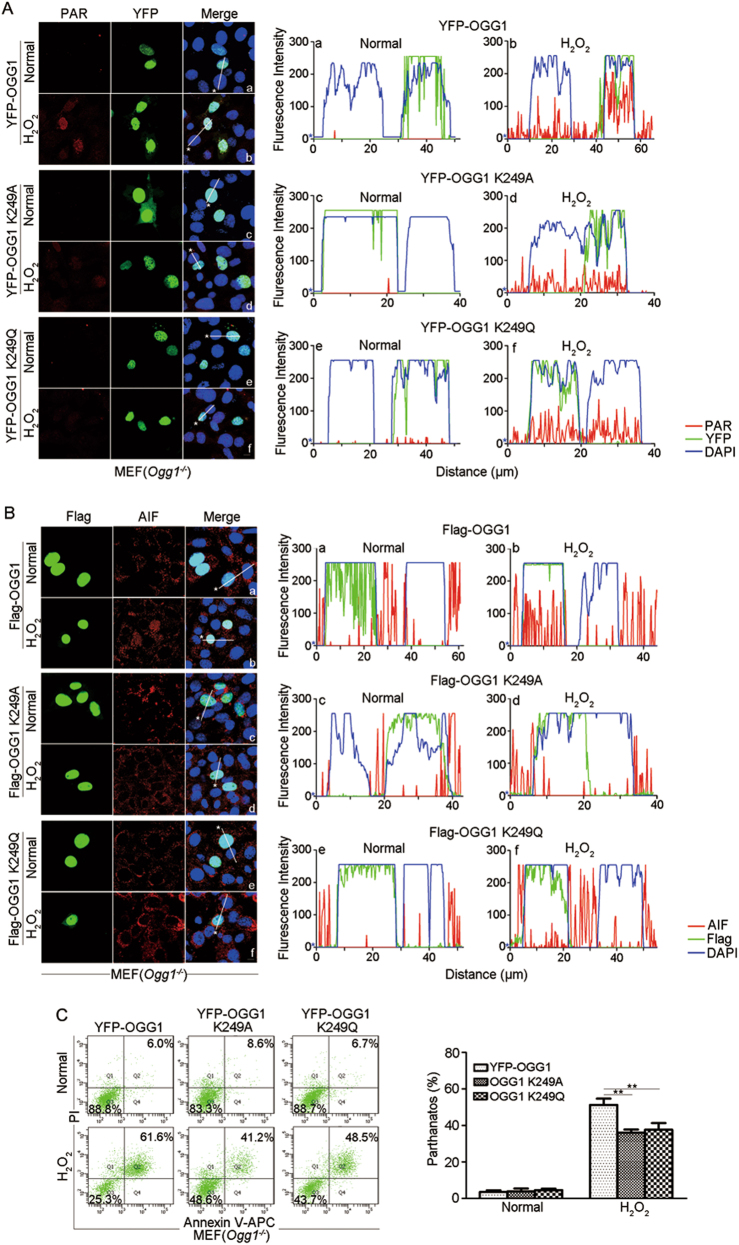
OGG1’s BER activity enhances AIF translocation to nuclei and parthanatos. **a** Cells expressing OGG1 mutants exhibit hampered protein PARylation in response to H_2_O_2_ exposure. YFP-OGG1, YFP-OGG1K249A, and YFP-OGG1K249Q plasmids were transfected into *Ogg1*^*−/−*^ MEFs for 36 h. Cells were incubated with H_2_O_2_ (400 μM) for 30 min. Immunofluorescence microscopy was performed to examine protein PARylation. Nuclei were counterstained with DAPI. The graphs (in the right panel) show the fluorescence intensity profiles in three fluorescence channels along line scans in the merged images (in the left panel), analyzed by Image Pro Plus software (red line: PARylation; green line: YFP; blue line: DAPI). **b**, **c** Cells expressing OGG1 K249 mutants exhibit hampered nuclear translocation of AIF and weakened parthanatos in response to H_2_O_2_ exposure. Transfection was conducted as described in **a**, and then **(b)** cells were incubated with H_2_O_2_ for 12 h. Microscopy was performed to visualize AIF and Flag. Nuclei were counterstained with DAPI. The right panels show the fluorescence intensity profiles as described in **a** (red line: AIF; green line: Flag; blue line: DAPI); or **c** cells were incubated with H_2_O_2_ for 24 h, and the YFP-positive cells were gated out. Cell death was examined by flow cytometry analysis of annexin V-APC/PI staining (left). Percentages of annexin-V and PI double-positive cells (parthanatos) were quantified and shown at the right panel. Scale bar: 10 μm. *n* ≥ 3; **p < 0.01

To investigate the role of OGG1-BER in parthanatos, plasmids carrying OGG1, OGG1K249A, and OGG1K249Q were transfected into *Ogg1*^*−/−*^ MEFs, respectively. It is worthwhile to note that excessive PARylation occurred in YFP-OGG1-expressing cells compared with those without YFP signals; whereas, the levels of PARylation in the YFP-OGG1K249A- and YFP-OGG1K249Q-expressing cells were weak and similar as that in YFP-negative cells (Fig. 5a). Immunofluorescence detection also revealed that in response to H_2_O_2_ exposure, nuclear AIF signals were overt in Flag-OGG1-expressing cells but not in those absent from Flag signals. Moreover, less AIF translocated into nuclei in the *Ogg1*^*−/−*^ MEFs expressing Flag-OGG1K249A or Flag-OGG1K249Q (Fig. 5b). Moreover, flow cytometry assay showed that incubation of H_2_O_2_ induced more YFP-OGG1-expressing cells undergoing parthanatos than the YFP-OGG1K249A- and YFP-OGG1K249Q-expressing ones (Fig. 5c). Taken together, these data indicated that OGG1-BER exacerbates oxidative stress-induced parthanatos.

### OGG1 is implicated in NMDA-induced neuronal cell parthanatos

**Fig. 6:**
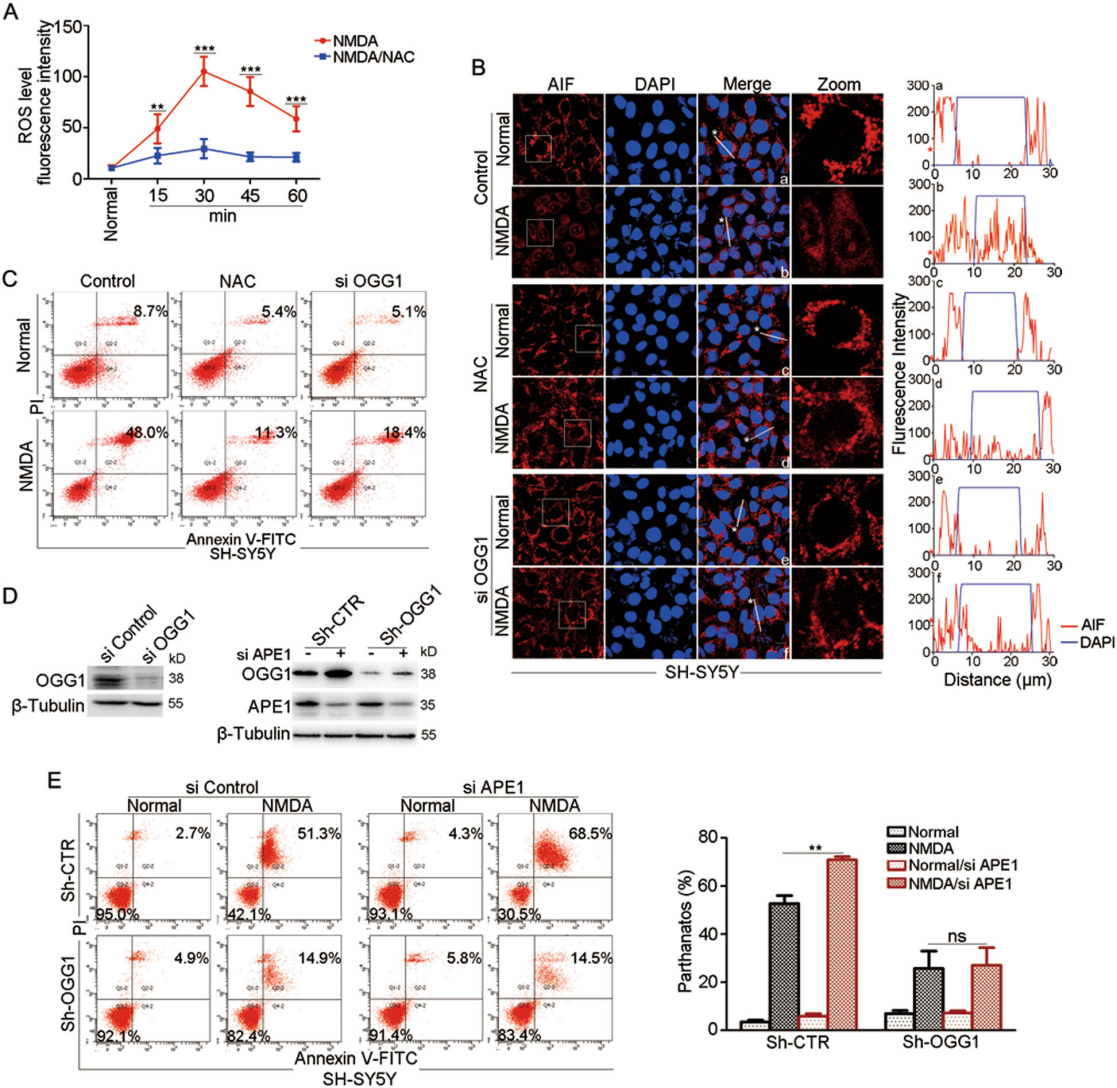
OGG1 is implicated in NMDA-induced neuroblastoma cell parthanatos. **a** NMDA exposure increases the level of intracellular ROS. Plasmids pHyper-Nuc were transfected into MEFs for 48 h, and cells were incubated with NMDA (500 μM) for the increasing lengths of times, as indicated in the presence or absence of 10 mM NAC. The average of GFP fluorescence intensity representing the nuclear ROS level was quantified using Image J software. Fifty cells were counted for each sample. **b** ROS scavenger or siRNA targeting OGG1 significantly hampered NMDA-induced nuclear translocation of AIF. SH-SY5Y cells were transfected with a siRNA against *OGG1* or control for 36 h. Cells were incubated with 500 μM NMDA for 30 min in the presence or absence of 10 mM NAC, and after 12 h, they were fixed and labeled with an antibody against AIF. The nuclei were counterstained with DAPI. Scale bar: 10 μm. The right column of the left panel shows magnified views of the boxed area in the AIF images. The profiles in the right panel show the fluorescence intensity of AIF from line scans in the merged images, which were analyzed by Image Pro Plus software (red line: AIF; blue line: DAPI). **c** ROS scavenger or siRNA targeting OGG1 significantly inhibited NMDA-induced cell death. SH-SY5Y cells were transfected with siRNA against OGG1 or control for 36 h. Cells were incubated with NMDA for 30 min in the presence or absence of 10 mM NAC, and after 24 h, cell death was examined by flow cytometry analysis of annexin V-FITC/PI staining. **d** Western blot detection of siOGG1 interference efficiency (left). Western blot analyses of Sh-OGG1 and siAPE1 interference efficiency in SH-SY5Ycells (right). **e** APE1 silencing has a deleterious effect on NMDA-induced cell death only with OGG1-proficient cells. Sh-CTR and Sh-OGG1 SH-SY5Y cells were transfected with siRNA against APE1 or control for 36 h. Cells were incubated with NMDA for 30 min, and after 24 h, cell death was examined by flow cytometry analysis of annexin V-FITC/PI staining (left). Percentages of annexin-V and PI double-positive cells (parthanatos) were quantified (right). *n* ≥ 3; **p < 0.01, ***p < 0.001

Parthanatos is a predominant form of neuron cell death triggered by the neurotoxicity of NMDA^28^. To verify the role of OGG1-BER in the induction of parthanatos, SH-SY5Y neuroblastoma cells were utilized. To determine the level of nuclear ROS induced by NMDA, pHyPer-Nuc GFP was overexpressed. Confocal microscopy detection revealed that NMDA exposure indeed increased the level of nuclear ROS, which was inhibited by NAC (Fig. 6a; Fig. S6). Thus, we proposed that OGG1-BER may enhance PARP1 overactivation and contribute to NMDA-induced neurons cell death. Immunofluorescence labeling showed that NMDA stimulation led to AIF translocation into the nucleus, which was hampered if the cells were pretreated with NAC or siRNA targeting OGG1 (Fig. 6b). Likewise, NMDA-induced cell death was also significantly lessened by administration of NAC or OGG1 silencing (Fig. 6c). The OGG1 interference efficiency was shown in Fig. 6d (left). These results taken together suggested that OGG1-BER may play an exacerbating role in NMDA-induced neurons parthanatos.

To further prove that DNA breaks left unrepaired after OGG1-BER play a major role in cell death induction, we detected the effects of OGG1 and APE1 double silencing. Recently, APE1 conditional knockout mouse was developed. The induced deletion of APE1 dramatically enlarged the infarct volume along with activation of PARP1 after transient focal cerebral ischemia^29^. Since APE1 primarily acts after glycosylases in BER pathway, we tested the effect of OGG1 expression on the consequence of APE1 deletion in the present study. To do so, we established OGG1 stably silenced SH-SY5Y cells as described under Materials and methods (Fig. 6d, right). OGG1-proficient or OGG1-deficient cells were further subjected to APE1 deletion or not, and then exposed to NMDA. In OGG1-proficient cells, APE1 deletion resulted in ~70% cells undergoing parthanatos upon NMDA treatment, which was ~20% higher than that of APE1-expressing cells; while OGG1 was deficient, cell death rate was only ~15%, and APE1 silencing did not further increase the deleterious effect (Fig. 6d, e). This result suggested an epistatic effect of OGG1 in cell death induction, and once OGG1 initiates the lesion excision, the repair intermediates may lead to hyperactivation of PARP1 and result in increased cell parthanatos if the follow-up steps in BER pathway are uncoupled.

## Discussion

The excessive ROS and resultant cell death have been well established as etiological factors involved in ischemic injury and degenerative alterations^10,30^. ROS cause damage to DNA, and the predominant form of DNA damages is base oxidation^4^. BER is a frontline defense to remove oxidized base lesions^2^. While it has been widely acknowledged that unrepaired DNA damages would lead to various deteriorated consequences^31,32^, our present study demonstrated that after oxidative insult, OGG1-initiated BER plays an exacerbating role in the induction of PARP1-dependent, AIF-mediated cell death, linking the process of base-excision repair to the pathogenesis of parthanatos.

Oxidized bases are the common oxidative damage along with other DNA lesions such as oxidized sugar fragments, abasic (AP) sites, and single-strand breaks (SSBs)^32,33^. Due to guanine’s lowest ionization potential among DNA bases^3^, the most predominant oxidative base product is 8-oxoG, which is primarily recognized and repaired by OGG1^5^. Repair of oxidized bases could further indirectly result in SSBs, and closely spaced SSBs may convert to DSBs^23^. Processing of oxidatively generated clustered DNA lesions may be prolonged, resulting in the increase in the persistent strand breaks^23^, which may account for the hyperactivation of PARP1 under oxidative stress.

The present study showed that the expression of OGG1 significantly augments cell death induced by a high level of H_2_O_2_ (Figs.1 and 2), whereas, lack of OGG1, even though resulting in sustained high level of 8-oxoG in genomic DNA (Fig. 4b), lowered H_2_O_2_-induced activation of PARP1 due to less level of DNA breaks including SSBs and DSBs (Fig. 3b–d). Likewise, less DNA breaks, decreased PARP1 activation, and AIF nuclear translocation were observed in OGG1K249A- and OGG1K294Q-expressing cells upon H_2_O_2_ exposure (Fig. 5a, b). On the contrary, while concentration of H_2_O_2_ was lower, OGG1 expression did not increase DNA strand breaks (Fig. 3a). Results collectively suggested that aggressive ROS may result in glycosylase-initiated BER overwhelming, and the successive strand break repair uncoupled; thus, the left-behind repair intermediates turn into major substrates for PARP1 activation.

The *Ogg1*^*−/−*^ mice were developed in two laboratories independently^34,35^. However, despite the accumulation of supraphysiological levels of 8-oxoG in the nuclear and mitochondrial genomes, the null animals displayed no noticeable changes in phenotype, including no impaired embryonic development, no marked pathological changes, or tumor induction^34–36^. Interestingly, homozygous deletion of nearly all of the DNA glycosylases is compatible with life^37,38^. In contrast, homozygous deletion of the core component of BER (such as APE1, XRCC1, and POL β) leads to early-stage embryonic or postnatal lethality^39,40^. The phenotypes of these null mice and the data of the present study suggested that once BER initiated, the uncoupled repair intermediates may evoke parthanatos, which accounts for the lethality of glycosylase genes deletion, whereas, the existence of oxidized bases is tolerable. In support, a study utilizing a transient focal cerebral ischemia model showed the activation of PARP1 and enlarged infarct volume in APE1 conditional knockout mouse^29^. And our result revealed that the deleterious effect of APE1 deletion in parthanatos induction only displayed in OGG1-proficient but not OGG1-deficient cells (Fig. 6e).

**Fig. 7:**
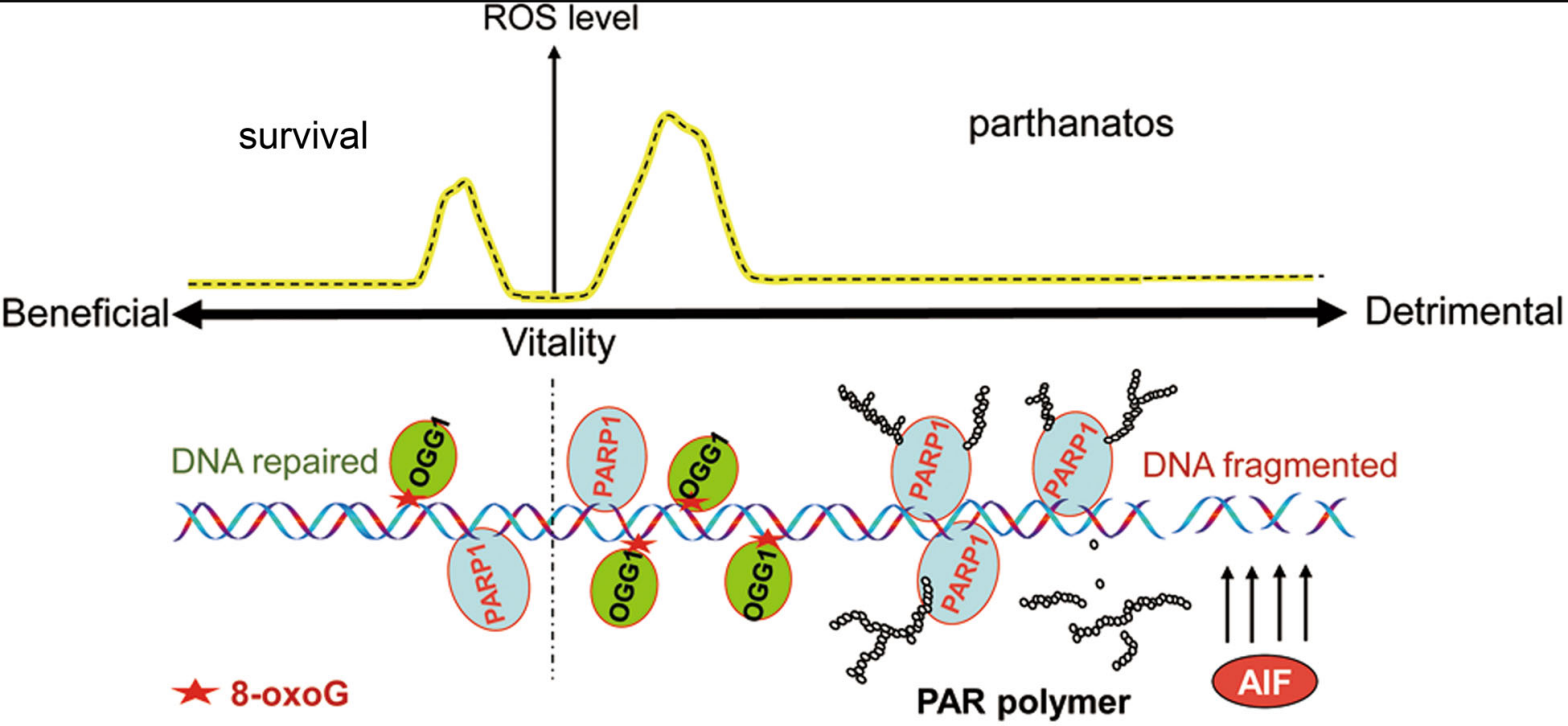
OGG1 functions as the guardian of the genome to remove the base lesion, and also facilitates cellular death process to maintain the homeostasis of the host. OGG1-initiated BER prevents accumulation of mutagenic guanine base lesions, and is beneficial for cell survival when oxidative insult is mild, and the level of 8-oxoG is low (left). However, if base oxidation is excessive, OGG1-BER causes strand break overwhelming and the repair uncoupled, which promotes PARP1 overactivation, free PAR formation and release, AIF transfer to the nucleus, and chromatin hydrolysis, leading to cell parthanatos (right)

Both OGG1 and PARP1 are responsible for DNA damage repair, and the cross talk between two types of enzymes has drawn attention^41^. Binding with and modification by PARP1 decreases the BER function of OGG1, which may suggest a feedback in turn ceasing OGG1-initiated BER-caused PARP1 overactivation^41^. There are studies documenting that no significant difference in cell survival was observed between wt and *Ogg1*^*−/−*^ cells^41^, and cells deficient in NTH1 or OGG1 exhibited elevated cell death after exposure of H_2_O_2_^42^. We deduced that the discrepancy of these conclusions from our studies may result from the mild extent of oxidative insults such as low concentrations or short exposure of H_2_O_2_, under which OGG1 expression does not increase DNA strand breaks (Fig. 3a), and glycosylase-initiated BER should be beneficial for cell survival (Fig. 7, left). But if base oxidation is excessive, OGG1-BER leads to strand break overwhelming and the repair uncoupled, which promotes PARP1 overactivation and cell death (Fig. 7, right).

Since our and others’ data^12,24,43,44^ demonstrated that H_2_O_2_-induced DNA damage effectively elicits PARP1 overactivation, ROS and glycosylase-initiated BER are implicated in *N*-methyl-d-aspartate (NMDA) and *N*-methyl-*N*′-nitro-*N*′-nitrosoguanidine (MNNG)-triggered cell death that has been widely taken as parthanatos, which arises as a question. Neuronal damage following stroke or in neurodegenerative diseases is thought to stem in part from overexcitation of NMDA receptors by glutamate, which results in parthanatos^22,45^. Excitotoxic activation of NMDA receptor results in formation of ROS and peroxynitrite (ONOO–), which inhibits cellular respiration, in turn leading to the positive feedback of increase in ROS generation. ROS cause DNA damage, which leads to the excessive PARP1 activation and AIF-mediated cell death (parthanatos)^14,22^. Our data showed that NMDA indeed elevated the intracellular ROS level in SH-SY5Y neuroblastoma cells (Fig. S6A, Fig. 6a), ROS scavenger and OGG1 interference significantly blocked AIF nuclear translocation and cell death (Fig. 6b, c). The alkylating agent MNNG induced oxidative stress and also caused excess DNA strand breaks that led to PARP1 overactivation and parthanatos^12,46^. Further study showed that antioxidant NAC abrogated all phenomena caused by MNNG, including enhanced DNA damage and PARP-1 activation^47^. It is possible that glycosylases such as OGG1 have a role in MNNG-induced parthanatos. All the studies suggested that ROS generation is a relevant event in pathological contexts, leading to DNA damage and then PARP1 overactivation. Glycosylase-initiated uncoupled BER exacerbates DNA damage, and thus it is a driving force for cells undergoing parthanatos.

Previous studies from our and others' groups presented that OGG1-BER activity is redox regulated^48–50^. When the intracellular context is oxidized, binding of enzymatically inactive OGG1 with promoter-located guanine lesion may be utilized to assemble the transcriptional machinery to launch the transcription of “ROS-response-ome”^49,51,52^. While redox balance is reestablished, OGG1 resumes its BER function. In the present study, the coincident kinetics of nuclear ROS and genomic 8-oxoG levels reflected an initiation of guanine lesion repair along with the reestablishment of the redox balance (Fig. 4a, b); however, the effective completion of the multiple-stepped BER somehow failed. Taken together, data suggested that while the redox levels orchestrate OGG1 to play a role either in gene transcription or in lesion repair; once BER is initiated after redox permission, the magnitude of base lesions, predominantly of 8-oxoG, defines the fate of cells, survival, or death.

In summary, our data link the repair of oxidized DNA bases to cell death triggered by PARP1 overactivation, and indicate that the OGG1-BER could be lethal for cells after exorbitant oxidative stress. Thus, the assessment of the prompt repair of oxidized bases under different conditions should be discrete. The present study suggested that OGG1 guards genome integrity not only depending on its capability to repair the base lesions, but also through promoting cell death to eliminate the cells with malignant potential, thus to maintain the homeostasis and protect the host.

## Materials and methods

### Antibodies and reagents

Rabbit anti-PAR polyclonal antibody (Ab) (4336-BPC-100; WB, 1:2000; IF, 1:200) was purchased from Trevigen (Gaithersburg, MD, USA). Mouse anti-8-oxoG monoclonal Ab (MOG100P; 1:1000) was from JaICA (Institute for the Control of Aging, Japan). Rabbit anti-Ogg1 polyclonal Ab with the specificity for recognition of mouse Ogg1 (ab135940; 1:3000) and monoclonal Ab with the specificity for recognition of human OGG1 (ab124741; 1:3000) were purchased from Abcam (San Francisco, CA). Mouse monoclonal anti-AIF (sc-13116; WB, 1:1000; IF, 1:200) and anti-APE1 (sc-17774; 1:2000) Abs were from Santa Cruz Biotechnology (Santa Cruz, CA, USA). Anti-γH2AX (9718S; 1:3000), rabbit polyclonal anti-AIF (4642S; IF, 1:200), and anti-caspase-3 (9662S; 1:3000) Abs were from Cell Signaling Technology (Boston, MA, USA). Anti-β-tubulin (HC101; 1:8000), anti-β-actin (HC201; 1:8000) mouse, and anti-GAPDH (H301; 1:5000) mouse monoclonal Abs were purchased from TRANS (Beijing, China). The monoclonal antibody against FLAG (F1804; IF, 1:500) was from Sigma (Saint Louis, MO, USA). Rabbit anti-lamin B polyclonal (Ab) (12987-1-AP; 1:3000) was purchased from Proteintech (Chicago, USA). Hydrogen peroxide (H_2_O_2_, 216763), PARP1 inhibitor PJ34 (P4365), N-methyl-D-aspartic acid (NMDA, M3262), and N-acetyl cystenic (NAC, A7250) and caspase-inhibitor VI (Z-VAD-FMK, 219007) were purchased from Sigma-Aldrich (St. Louis, MO, USA).

### Cell culture and treatments

Wt, *Ogg1*^*−/−*^, and *Nth1*^*−/−*^ MEFs were kindly provided by Dr. Istvan Boldogh (University of Texas Medical Branch at Galveston, Texas). SH-SY5Y neuroblastoma cells were kindly provided by Dr. Pengfei Ge (Department of Neurosurgery, First Hospital of Jilin University, Changchun, China). Cells were grown in DMEM (Invitrogen) supplemented with 10% fetal bovine serum. For the induction of cell death, cells were incubated with indicated concentrations of H_2_O_2_ for 24 h. For detection of protein PARylation, cells were incubated with H_2_O_2_ for 30 min. Alternatively, cells were incubated with H_2_O_2_ for 30 min or 24 h in the presence or absence of the following inhibitors: PJ34 (2.5 μM) or NAC (10 mM).

### Plasmids and transfection

YFP-OGG1, Flag-OGG1 plasmids were constructed by inserting the OGG1 coding sequence into pZsYellow1-C1, pcDNA3.1(+) vectors in our laboratory. Transfection was performed using Lipofectamine 3000 reagent (Invitrogen, Carlsbad, CA) according to the manufacturer’s instructions. Cells were used for the experiment 36 h post transfection.

### SiRNA-mediated interference

For RNA interference experiments, scramble control, or smart-pool siRNA respectively against hOGG1 and mOgg1 (Dharmacon), APE1 and AIF were transfected into MEFs or SH-SY5Y cells using Lipofectamine 3000 according to the manufacturer’s instructions. siRNA duplex oligoribonucleotides were synthesized by GenePharma (Shanghai, China). The sense sequences were as follows: control siRNA (nonsense oligo), 5′-UUCUCCGAACGUGUCACGUTT-3′; AIF siRNA, 5′-GAAACUGACCACAUAGUGATT-3′; and APE1 siRNA, 5′-GUCUGGUACGACUGGAGUACC-3′. Cells were used for experiments after 48 h of interference, and the efficacy of target gene knockdown was validated by western blotting.

### ShRNAs and lentiviral transduction

To stably knock down OGG1 in SH-SY5Y cells, the pLKO.1-TRC cloning vector (Addgene) was used. The OGG1 shRNA sequences were 5′-CCGGAAAGAGGTGGCTCAGAAATTCCTCGAGGAATTTCTGAGCCACCTCTTTTTTTTG-3′ and 5′- AATTCAAAAAAAAGAGGTGGCTCAGAAATTCCTCGAGGAATTTCTGAGCCACCTCTTT-3′. The shRNAs were synthesized and cloned into the vectors. The constructed plasmids and shCtrl plasmid were transfected into 293T cells, together with the packaging plasmid psPAX2 and the envelope plasmid pMD2.G (both from Addgene) by using the Lipofectamine 2000 reagent (Invitrogen). And the collection of supernatant and 5 mg/ml polybrene (Sigma) were used to infect the cells. The cells were selected in puromycin-containing medium 3 days after infection.

### Immunofluorescence microscopy

Cells grown on glass coverslips were fixed with 10% (v/v) formaldehyde in PBST (PBS with 0.1% Triton X-100) for 10 min, permeabilized with 0.5% (v/v) Triton X-100 for 5 min, and then blocked with 10% (w/v) bovine serum albumin for 30 min. Thereafter, cells were incubated with the primary Abs recognizing PAR (1:200), AIF (1:200), Flag (1:500), and then the secondary Abs successively. The nuclei of the cells were stained with 4,6-diamidino-2-phenylindole (DAPI) for 5 min. Cells were visualized by using a confocal microscope (Nikon, Tokyo, Japan) equipped with a ×60 oil-immersion objective lens. The total protein PARylation was quantified by using Image J software. Fluorescence intensity of proteins (PARylation, AIF, YFP, Flag, and DAPI) was quantified by using IPP software.

To quantify the amount of nuclear AIF, Image J software was used. First, the total AIF amount was measured, and then measure the AIF amount of nuclear. The transfer of AIF was quantified by the nuclear divided by the total amount of AIF.

### Cell fractionation

Collection of cell fractionations was performed as described previously^53^, with slight modifications. Briefly, for western blot, cells were harvested, washed with PBS, and lysed in RIPA buffer (50 mM Tris-HCl, pH 7.4, 150 mM NaCl, 1% NP-40, 1 mM EDTA, 1 mM Na_3_VO_4_, 2 mM NaF, 1 mM β-glycerphosphate, and 2.5 mM sodium pyrophosphate containing 1 mM PMSF and protease inhibitors) for 30 min on ice. Lysates were centrifuged at 12,000 × *g* for 25 min at 4 °C, and the supernatant was the whole-cell extract (WE). Cytoplasmic and nuclear fractions were prepared using the CelLytic NuCLEAR Extraction Kit (Sigma) following the manufacturer’s instruction. Briefly, cells were lysed with cytosolic lysis buffer for 20 min, lysates were centrifuged at 11,000 × *g* for 1 min at 4 °C, and the supernatant was the cytoplasmic extract (CE). The pellet was washed twice with cytosolic lysis buffer and lysed with nuclear extraction buffer on ice for 30 min. Nuclear lysates were centrifuged at 21,000 × *g* for 5 min at 4 °C and the supernatant was the nuclear extract (NE).

### Western blotting

MEFs and SH-SY5Y cells were stimulated and lysed. WE, CE, and NE, prepared as described above, were mixed with SDS sample buffer, separated by 10% SDS-PAGE, and transferred onto nitrocellulose membranes. Membranes were blocked with TBS containing 0.1% Tween 20 (TBST) and 5% skim milk for 1 h and incubated with primary Abs overnight. After three times of washing with TBST, membranes were incubated with secondary Abs and detected by ECL Plus western blot detection reagents.

### Flow cytometry

Annexin V-FITC (AO2001-10) and Annexin V-APC (AO2001-11) Apoptosis Analysis Kits (Sungene Biotech, Tianjin, China) were used to detect cell apoptosis. Cell death detection was performed following the manufacturer’s instruction. Briefly, cells were harvested, washed with cold PBS, suspended in 1× binding buffer, and then centrifuged at 300 × *g* for 10 min. Supernatants were removed, and cells were resuspended in 100 μL of binding buffer with addition of 5 μL of annexin V-FITC (APC), and after a gentle vortex, they were incubated for 10 min in room temperature, and then another 5 min in room temperature after addition of 5 μL of PI solution. Flow cytometry was performed by using a FACSSanto II (BD Biosciences), and data were analyzed with FACSDiva software (BD Biosciences).

### Comet assay

Alkaline, neutral comet assays were performed using the Comet Assay Kit from Trevigen (Gaithersburg, MD) following the manufacturer’s guidance. Images were captured using the fluorescent microscope (ECLIPSE, 80i, Nikon, Japan). Tail moment was determined using Cometscore software (TriTek, Sumerduck, USA).

### Dot-blot analysis

Dot-blot analysis was performed as described previously^54^, with slight modifications. Genomic DNA was isolated using a TIANamp Genomic DNA Kit (TIANGEN, Beijing, China). RNase A (Sigma) digestion was included in the isolation procedure. Isolated genomic DNA (1 μg per sample) was denatured (0.4 M NaOH and 10 mM EDTA) at 95 °C for 5 min, and chilled immediately on ice for 10 min. Samples were serially twofold diluted and spotted on nitrocellulose membrane using the BioDot Microfiltration Apparatus (170-6545, Bio-Rad). The blotted membrane was cross-linked under UV for 4 min, and incubated in 2× SSC for 5 min. The membrane was blocked with TBS containing 0.1% Tween 20 (TBST) and 5% skim milk for 1 h and incubated with anti-8-oxoG (1:1000) Ab overnight, and after three times of washing with TBST, it was incubated with secondary Ab and detected by ECL Plus western blot detection reagents.

### TUNEL labeling

The YF594 TUNEL Assay Apoptosis Detection Kit (US Everbright Inc.) was used to label 3′-OH of DNA breaks following the manufacturer’s guidance. Data were analyzed by FACSDiva (BD Biosciences) and FlowJo7.6.1 software.

### Nuclear ROS assay

To assess the changes in nuclear ROS level, the hydrogen peroxide sensor pHyper-Nuc GFP (Evrogen, Axxora Inc., Farmingdale, NY) was used^55^. Cells grown in 35-mm dishes with 14-mm glass bottoms were tranfected with pHyPer-Nuc GFP (nuclear localization signal fused to the C-terminus of Hyper) for 48 h, treated with 400 μM H_2_O_2_, and placed on a 37 °C heated stage with 5% CO_2_ atmosphere chamber. The sequential images were captured by using an UltraVIEW Vox (PerkinElmer Inc., Waltham, MA) spinning-disk confocal microscope with a Ti-E microscope (Nikon, Japan) in fluorescence channel at 60-s intervals over a 60-min timecourse using a ×20 objective.

To detect nuclear ROS level in SH-SY5Y cells, pHyper-Nuc GFP was transfected into SH-SY5Y cells for 48 h. Cells were treated with NMDA (500 μM) for the increasing lengths of time (0, 15, 30, 45, and 60 min). Then they were fixed with 10% (v/v) formaldehyde in PBST (PBS with 0.1% Triton X-100) for 10 min, permeabilized with 0.5% (v/v) Triton X-100 for 5 min, and then blocked with 10% (w/v) bovine serum albumin for 30 min. Cells were visualized by using a confocal microscope (Nikon, Tokyo, Japan) equipped with a ×60 oil-immersion objective lens. The relative GFP fluorescence intensity were quantified by using Image J software.

### Statistical analysis

The data were analyzed using Student’s *t*-test and presented as the means ± SEM. Quantifications are based on at least three independent experiments. The level of significance was accepted at *P < 0.05, **P < 0.01, and ***P < 0.001

## Electronic supplementary material

Supplementary figure file

Supplementary Figure 1,2,3

Supplementary Figure 4,5,6

## References

[1] Schieber M, Chandel NS (2014). ROS function in redox signaling and oxidative stress. Curr. Biol..

[2] David SS, O’Shea VL, Kundu S (2007). Base-excision repair of oxidative DNA damage. Nature.

[3] Barnes DE, Lindahl T (2004). Repair and genetic consequences of endogenous DNA base damage in mammalian cells. Annu. Rev. Genet..

[4] Lindahl T, Barnes DE (2000). Repair of endogenous DNA damage. Cold Spring Harb. Symp. Quant. Biol..

[5] Wallace SS (2013). DNA glycosylases search for and remove oxidized DNA bases. Environ. Mol. Mutagen..

[6] Dizdaroglu M, Kirkali G, Jaruga P (2008). Formamidopyrimidines in DNA: mechanisms of formation, repair, and biological effects. Free Radic. Biol. Med..

[7] Krokan HE, Bjoras M (2013). Base excision repair. Cold Spring Harb. Perspect. Biol..

[8] Izumi T (2003). Mammalian DNA base excision repair proteins: their interactions and role in repair of oxidative DNA damage. Toxicology.

[9] Odell ID, Wallace SS, Pederson DS (2013). Rules of engagement for base excision repair in chromatin. J. Cell. Physiol..

[10] Cross CE (1987). Oxygen radicals and human disease. Ann. Intern. Med..

[11] Fulda S, Gorman AM, Hori O, Samali A (2010). Cellular stress responses: cell survival and cell death. Int. J. Cell Biol..

[12] Yu SW (2002). Mediation of poly(ADP-ribose) polymerase-1-dependent cell death by apoptosis-inducing factor. Science.

[13] Yu SW (2006). Apoptosis-inducing factor mediates poly(ADP-ribose) (PAR) polymer-induced cell death. Proc. Natl Acad. Sci. USA.

[14] Wang Y, Dawson VL, Dawson TM (2009). Poly(ADP-ribose) signals to mitochondrial AIF: a key event in parthanatos. Exp. Neurol..

[15] Khodyreva SN (2010). Apurinic/apyrimidinic (AP) site recognition by the 5’-dRP/AP lyase in poly(ADP-ribose) polymerase-1 (PARP-1). Proc. Natl Acad. Sci. USA.

[16] Wang Z, Wang F, Tang T, Guo C (2012). The role of PARP1 in the DNA damage response and its application in tumor therapy. Front. Med..

[17] Luo X, Kraus WL (2012). On PAR with PARP: cellular stress signaling through poly(ADP-ribose) and PARP-1. Genes Dev..

[18] Wang Y (2011). Poly(ADP-ribose) (PAR) binding to apoptosis-inducing factor is critical for PAR polymerase-1-dependent cell death (parthanatos). Sci. Signal..

[19] Pacher P, Szabo C (2008). Role of the peroxynitrite-poly(ADP-ribose) polymerase pathway in human disease. Am. J. Pathol..

[20] Wang H, Shimoji M, Yu SW, Dawson TM, Dawson VL (2003). Apoptosis inducing factor and PARP-mediated injury in the MPTP mouse model of Parkinson’s disease. Ann. N. Y. Acad. Sci..

[21] Ba X (2014). The role of 8-oxoguanine DNA glycosylase-1 in inflammation. Int. J. Mol. Sci..

[22] Dawson VL, Dawson TM (2004). Deadly conversations: nuclear-mitochondrial cross-talk. J. Bioenerg. Biomembr..

[23] Georgakilas AG (2008). Processing of DNA damage clusters in human cells: current status of knowledge. Mol. Biosyst..

[24] Batnasan E (2015). 17-Beta estradiol inhibits oxidative stress-induced accumulation of AIF into nucleolus and PARP1-dependent cell death via estrogen receptor alpha. Toxicol. Lett..

[25] Virag L, Szabo C (2002). The therapeutic potential of poly(ADP-ribose) polymerase inhibitors. Pharmacol. Rev..

[26] Jamil S, Lam I, Majd M, Tsai SH, Duronio V (2015). Etoposide induces cell death via mitochondrial-dependent actions of p53. Cancer Cell Int..

[27] Bruner SD, Norman DP, Verdine GL (2000). Structural basis for recognition and repair of the endogenous mutagen 8-oxoguanine in DNA. Nature.

[28] Yu SW, Wang H, Dawson TM, Dawson VL (2003). Poly(ADP-ribose) polymerase-1 and apoptosis inducing factor in neurotoxicity. Neurobiol. Dis..

[29] Stetler RA (2016). APE1/Ref-1 facilitates recovery of gray and white matter and neurological function after mild stroke injury. Proc. Natl Acad. Sci. USA.

[30] Gorenkova N, Robinson E, Grieve DJ, Galkin A (2013). Conformational change of mitochondrial complex I increases ROS sensitivity during ischemia. Antioxid. Redox Signal..

[31] Kidane D (2014). Interplay between DNA repair and inflammation, and the link to cancer. Crit. Rev. Biochem. Mol. Biol..

[32] Hegde ML, Izumi T, Mitra S (2012). Oxidized base damage and single-strand break repair in mammalian genomes: role of disordered regions and posttranslational modifications in early enzymes. Prog. Mol. Biol. Transl. Sci..

[33] Hegde ML, Hazra TK, Mitra S (2008). Early steps in the DNA base excision/single-strand interruption repair pathway in mammalian cells. Cell Res..

[34] Klungland A (1999). Accumulation of premutagenic DNA lesions in mice defective in removal of oxidative base damage. Proc. Natl Acad. Sci. USA.

[35] Minowa O (2000). Mmh/Ogg1 gene inactivation results in accumulation of 8-hydroxyguanine in mice. Proc. Natl Acad. Sci. USA.

[36] Arai T, Kelly VP, Minowa O, Noda T, Nishimura S (2006). The study using wild-type and Ogg1 knockout mice exposed to potassium bromate shows no tumor induction despite an extensive accumulation of 8-hydroxyguanine in kidney DNA. Toxicology.

[37] Cabelof DC (2012). Haploinsufficiency in mouse models of DNA repair deficiency: modifiers of penetrance. Cell. Mol. Life Sci..

[38] Sampath H (2014). Oxidative DNA damage in disease--insights gained from base excision repair glycosylase-deficient mouse models. Environ. Mol. Mutagen..

[39] Brenerman BM, Illuzzi JL, Wilson DM (2014). Base excision repair capacity in informing healthspan. Carcinogenesis.

[40] Abbotts R, Wilson DM (2017). Coordination of DNA single strand break repair. Free Radic. Biol. Med..

[41] Noren Hooten N, Kompaniez K, Barnes J, Lohani A, Evans MK (2011). Poly(ADP-ribose) polymerase 1 (PARP-1) binds to 8-oxoguanine-DNA glycosylase (OGG1). J. Biol. Chem..

[42] Yang N, Chaudhry MA, Wallace SS (2006). Base excision repair by hNTH1 and hOGG1: a two edged sword in the processing of DNA damage in gamma-irradiated human cells. DNA Repair (Amst.).

[43] Xu H (2013). Iduna protects HT22 cells from hydrogen peroxide-induced oxidative stress through interfering poly(ADP-ribose) polymerase-1-induced cell death (parthanatos). Cell. Signal..

[44] Zheng L (2017). JNK activation contributes to oxidative stress-induced parthanatos in glioma cells via increase of intracellular ROS production. Mol. Neurobiol..

[45] Wang, Y. et al. A nuclease that mediates cell death induced by DNA damage and poly(ADP-ribose) polymerase-1. *Science* **354, **aad6872 (2016). 10.1126/science.aad6872 PMC5134926 27846469

[46] Artus C (2010). AIF promotes chromatinolysis and caspase-independent programmed necrosis by interacting with histone H2AX. EMBO J..

[47] Chiu LY, Ho FM, Shiah SG, Chang Y, Lin WW (2011). Oxidative stress initiates DNA damager MNNG-induced poly(ADP-ribose)polymerase-1-dependent parthanatos cell death. Biochem. Pharmacol..

[48] Bravard A (2006). Redox regulation of human OGG1 activity in response to cellular oxidative stress. Mol. Cell. Biol..

[49] Pan L (2016). Oxidized guanine base lesions function in 8-oxoguanine DNA glycosylase-1-mediated epigenetic regulation of nuclear factor kappaB-driven gene expression. J. Biol. Chem..

[50] Bravard A (2010). Inactivation by oxidation and recruitment into stress granules of hOGG1 but not APE1 in human cells exposed to sub-lethal concentrations of cadmium. Mutat. Res..

[51] Ba X (2014). 8-Oxoguanine DNA glycosylase-1 augments proinflammatory gene expression by facilitating the recruitment of site-specific transcription factors. J. Immunol..

[52] Ba X, Boldogh I (2018). 8-Oxoguanine DNA glycosylase 1: beyond repair of the oxidatively modified base lesions. Redox Biol..

[53] Ke Y (2017). PARP1 promotes gene expression at the post-transcriptiona level by modulating the RNA-binding protein HuR. Nat. Commun..

[54] Pan L (2017). OGG1-DNA interactions facilitate NF-kappaB binding to DNA targets. Sci. Rep..

[55] Hajas G (2013). 8-Oxoguanine DNA glycosylase-1 links DNA repair to cellular signaling via the activation of the small GTPase Rac1. Free Radic. Biol. Med..

