# Supplementary material for: OGG1-initiated base excision repair exacerbates oxidative stress-induced parthanatos
Source: Cell Death Dis. 2018 May 24;9(6):628. doi: 10.1038/s41419-018-0680-0 (PMC5967321; doi:10.1038/s41419-018-0680-0)
Supplement: Supplementary file 1 — Supplementary figure file [file 41419_2018_680_MOESM1_ESM.docx]

**Supplementary Figure legends**

**Supplementary Figure 1. PJ34 inhibits H_2_O_2_-induced PARP1 activation.**

(A) H_2_O_2_ exposure increases levels of protein PARylation. MEFs were incubated with increasing concentrations of H_2_O_2_ for 30 min and then harvested. Whole cell lysates were prepared, and western blotting was performed to examine the changes in levels of protein PARylation. (B) MEFs were incubated with 400 μM H_2_O_2_ in the presence of 10 μM PJ34 or not. Western blot was performed to examine protein PARylation.

**Supplementary Figure 2. ETO exposure elicits cell death without induction of PARP1 activation.**

(A) ETO exposure elicits cell death in a dose-dependent manner. MEFs cells were incubated with increasing concentrations of ETO for 24 h. Cell death was examined by flow cytometry analysis of Annexin V-FITC/PI staining. (B) Microscopic assessment of protein PARylation. MEFs were incubated with increasing concentrations of ETO for 30 min and then fixed. Immunofluorescence microscopy was performed to examine protein PARylation. Nuclei were counter-stained with DAPI. Scale bar: 10 μm.

**Supplementary Figure 3. Caspase-3 is not activated during H_2_O_2_-induced cell death.**

MEFs were exposed to 400 μM H_2_O_2_ for the increasing lengths of time as indicated, and 300 μM ETO for 12h as a positive control. Whole cell lysates were prepared, and western blotting was performed to examine cleavage of caspase-3.

**Supplementary Figure 4. *Nth1* deletion prevents cells undergoing cell death upon H_2_O_2_ exposure.** *Nth1^+/+^* and *Nth1^-/-^* MEFs were incubated with 400 μM H_2_O_2_ for 24 h. Cell death was examined by flow cytometry analysis of Annexin V-FITC/PI staining.

**Supplementary Figure 5. OGG1 augments DNA DSB response and promotes AIF transfer to nucleus in cells exposed to H_2_O_2_**.

(A) Less DSB response is evoked in *Ogg1*-null cells upon H_2_O_2_ exposure. *Ogg1^+/+^* and *Ogg1^−/−^* MEFs were exposed to 400 μM H_2_O_2_ for the increasing lengths of time as indicated. Whole cell lysates were prepared, and western blotting was performed to examine the level of γH2AX.

(B) OGG1 promotes nuclear translocation of AIF in response to H_2_O_2_ exposure. *Ogg1^+/+^* and *Ogg1^−/−^* MEFs were incubated with H_2_O_2_ for increasing lengths of time as indicated, and then the cells were harvested, cytoplasmic and nuclear fractions were prepared. AIF levels were analyzed by western blotting. Lamin B was taken as the nuclear control and GAPDH was as the cytoplasmic.

**Supplementary Figure 6.** **NMDA exposure enhances the level of nuclear ROS.**

Nuclear ROS sensor pHyper-Nuc GFP were transfected into SH-SY5Y cells for 48h. Cells were treated with NMDA (500μM) in the presence or absence of NAC for the increasing lengths of time as indicated, and then fixed. Confocal microscopy was performed to visualize GFP signals. Scale bar: 10 μm.
