# Supplementary figures and images for: OGG1-initiated base excision repair exacerbates oxidative stress-induced parthanatos
Source: Cell Death Dis. 2018 May 24;9(6):628. doi: 10.1038/s41419-018-0680-0 (PMC5967321; doi:10.1038/s41419-018-0680-0)

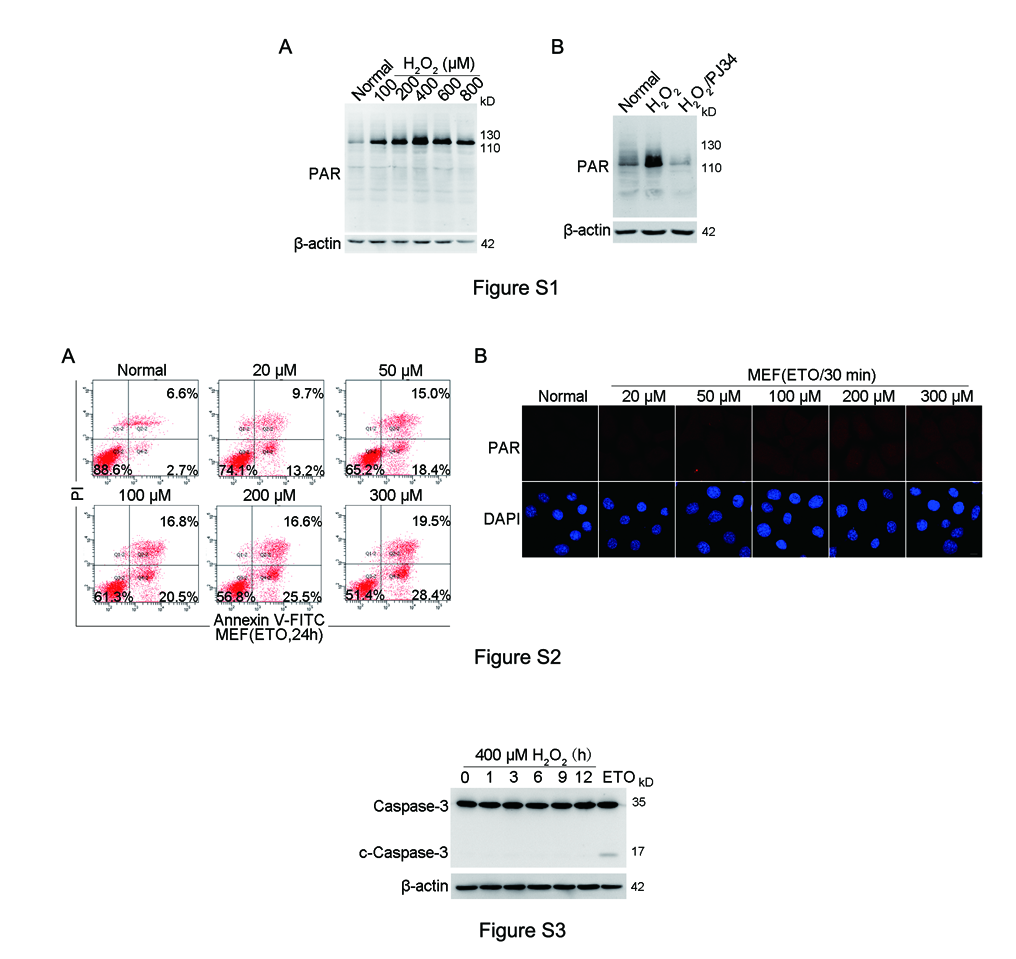

Supplement: Supplementary file 2 — Supplementary Figure 1,2,3 [file 41419_2018_680_MOESM2_ESM.tif]

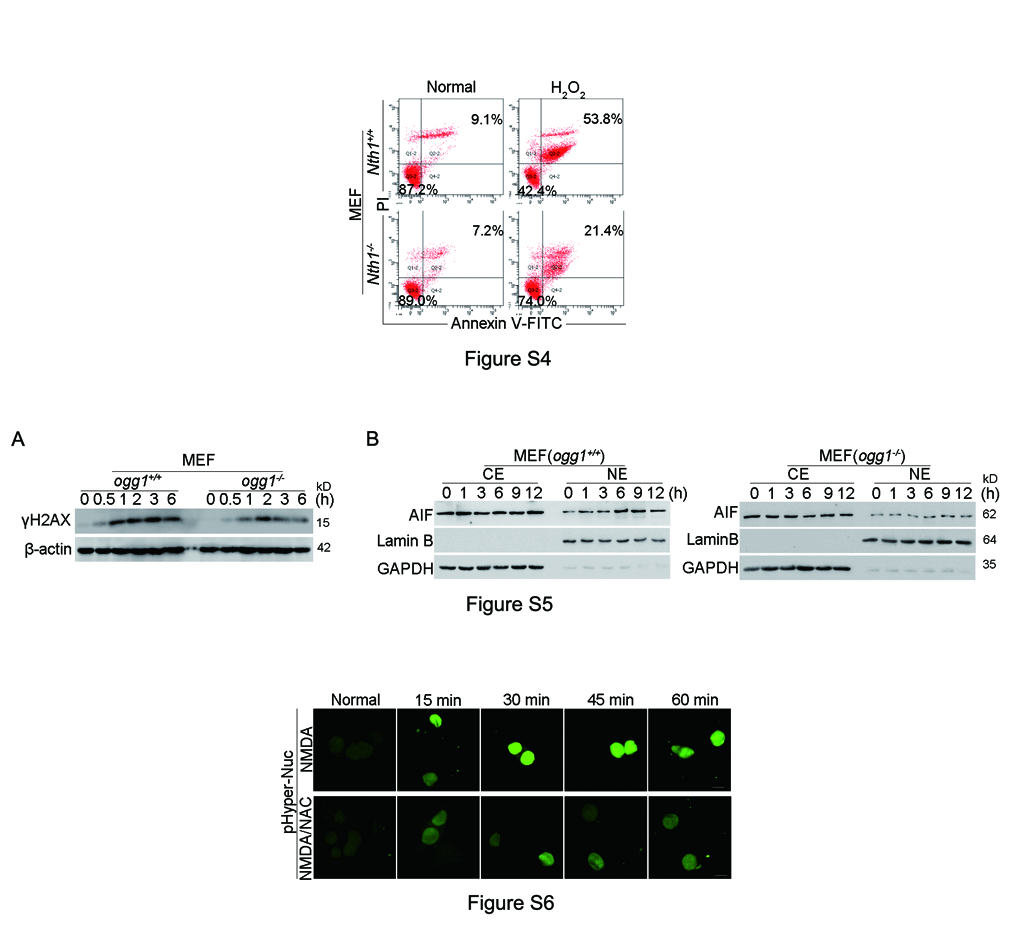

Supplement: Supplementary file 3 — Supplementary Figure 4,5,6 [file 41419_2018_680_MOESM3_ESM.tif]
